# Accurate Prostate Segmentation in Large-Scale Magnetic Resonance Imaging Datasets via First-in-First-Out Feature Memory and Multi-Scale Context Modeling

**DOI:** 10.3390/jimaging11020061

**Published:** 2025-02-16

**Authors:** Jingyi Zhu, Xukun Zhang, Xiao Luo, Zhiji Zheng, Kun Zhou, Yanlan Kang, Haiqing Li, Daoying Geng

**Affiliations:** 1 Academy for Engineering and Technology, Fudan University, Shanghai 200433, China; 21110860056@m.fudan.edu.cn (J.Z.); zhangxk21@m.fudan.edu.cn (X.Z.); luox19@fudan.edu.cn (X.L.); 23110860044@m.fudan.edu.cn (Z.Z.); 23110860035@m.fudan.edu.cn (K.Z.); 21110860035@m.fudan.edu.cn (Y.K.); 2 Department of Radiology, Huashan Hospital, Fudan University, Shanghai 200400, China; haiqingli@fudan.edu.cn

**Keywords:** prostate segmentation, context modeling module, dynamic adjustment mechanism, T2-weighted imaging

## Abstract

Prostate cancer, a prevalent malignancy affecting males globally, underscores the critical need for precise prostate segmentation in diagnostic imaging. However, accurate delineation via MRI still faces several challenges: (1) The distinction of the prostate from surrounding soft tissues is impeded by subtle boundaries in MRI images. (2) Regions such as the apex and base of the prostate exhibit inherent blurriness, which complicates edge extraction and precise segmentation. The objective of this study was to precisely delineate the borders of the prostate including the apex and base regions. This study introduces a multi-scale context modeling module to enhance boundary pixel representation, thus reducing the impact of irrelevant features on segmentation outcomes. Utilizing a first-in-first-out dynamic adjustment mechanism, the proposed methodology optimizes feature vector selection, thereby enhancing segmentation outcomes for challenging apex and base regions of the prostate. Segmentation of the prostate on 2175 clinically annotated MRI datasets demonstrated that our proposed MCM-UNet outperforms existing methods. The Average Symmetric Surface Distance (ASSD) and Dice similarity coefficient (DSC) for prostate segmentation were 0.58 voxels and 91.71%, respectively. The prostate segmentation results closely matched those manually delineated by experienced radiologists. Consequently, our method significantly enhances the accuracy of prostate segmentation and holds substantial significance in the diagnosis and treatment of prostate cancer.

## 1. Introduction

Prostate cancer is the second most frequently diagnosed cancer in men and the fifth leading cause of death worldwide [1]. The annual incidence of prostate cancer has increased in recent years. Predictions suggest that the annual number of new cases will rise from 1.4 million in 2020 to 2.9 million by 2040 [2]. The early detection and risk assessment of prostate cancer are crucial for effective treatment planning and for improving patient outcomes [3]. Common diagnostic methods for prostate cancer include digital rectal examination (DRE) and prostate-specific antigen (PSA) testing, with a definitive diagnosis typically confirmed by prostate biopsy [4]. However, these tests often cause physical discomfort to patients. Magnetic resonance imaging (MRI) has become a crucial method for detecting prostate cancer, as it offers clear anatomical images. MRI encompasses various imaging modalities, including T2-weighted (T2W), diffusion-weighted imaging (DWI), and dynamic contrast-enhanced (DCE) imaging. External beam radiation therapy (EBRT) is a common treatment modality for prostate cancer [5,6]. Precise MRI segmentation of the prostate is essential for effective prostate cancer management, enabling accurate radiation therapy and minimizing radiation-related damage to the surrounding healthy tissues [7].

**Figure 1:**
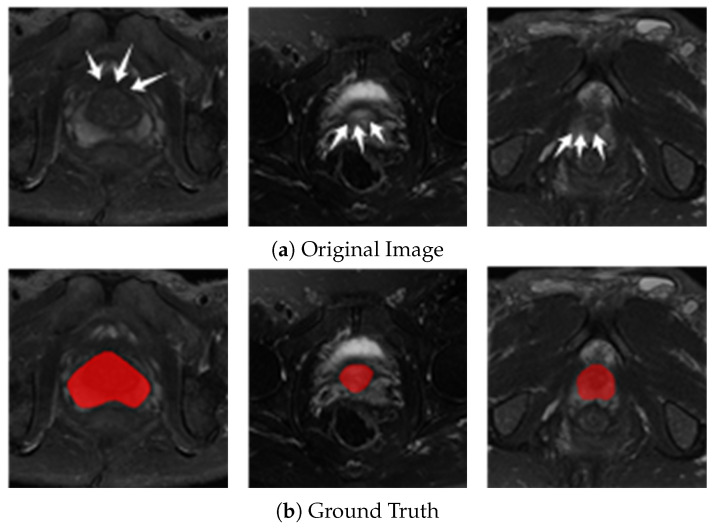
Challenges in automated prostate segmentation in T2W images. This figure illustrates the primary challenges in the automated segmentation of the prostate in T2W images. Displayed are the original image sequence and the corresponding reference standard for a specific instance. In the reference images, white arrows highlight areas of the prostate with vague and irregular borders. The prostate regions are segmented in red.

However, achieving accurate delineation using MRI presents several challenges. First, the boundaries between the prostate and surrounding soft tissues are often ambiguous in MRI images, leading to time-consuming manual annotations that are prone to inter-operator variability. Second, the apex and base of the prostate inherently display blurriness, which complicates edge extraction and precise segmentation [8]. Figure 1 shows a T2W sequence image of the prostate, with the red area indicating the prostate region. As illustrated in Figure 1, the boundaries of the prostate tissue are notably blurred, particularly in the apex and base regions. This blurriness significantly complicates the automatic segmentation.

With the rapid advancement of artificial intelligence and computer vision, these technologies have found wide-spread application in medical image processing. Given the distinct characteristics of natural and medical images, particularly prostate MRI scans, applying deep learning algorithms to localize and segment these images is crucial to enhance the accuracy and efficiency of prostate cancer diagnosis [9]. Numerous medical image segmentation techniques based on traditional machine learning have been extensively investigated, including atlas-based approaches [10], graph-cut algorithms [11], and watershed transformations [12]. These methods have been employed for the quantitative assessment of regions within various medical images. However, most of these techniques rely on manually constructed features that do not effectively capture the robust visual cues required to overcome the challenges inherent in the segmentation task. Since 2012, radiomics has emerged as a complementary approach to medical image analysis, extracting a large number of quantitative features, such as texture, shape, and intensity, from medical images. These features have been used to predict clinical outcomes and disease characteristics more effectively [13,14].

In recent years, deep learning, particularly convolutional neural networks (CNN) [15], has become increasingly prevalent in medical image segmentation and has demonstrated remarkable success. Foundational models such as the Fully Convolutional Network (FCN) [16], U-Net [17], and Residual Networks (ResNet) [18] have achieved significant milestones in the domain of medical image segmentation. In line with this, the dilated One-to-Many U-Net model has been proposed to address segmentation challenges posed by diverse imaging modalities and varied target sizes, achieving impressive results on datasets such as the HC18 ultrasound dataset and the Multi-site MRI dataset, with Dice coefficients of 96.54% and 96.76% for fetal head and prostate segmentation, respectively [19]. Among them, nnU-Net [20,21], the most widely recognized convolutional network-based model, has proven to be suitable for most medical segmentation tasks. U-Net++ [22] utilizes more nested and densely connected skip connections to better capture the fine-grained details of foreground objects [23]. Although models based on U-Net [17] have significantly advanced medical segmentation, they often struggle to capture long-range relationships and global contextual information due to the limited receptive field of the convolutional kernels. Consequently, researchers have shifted their focus to self-attention mechanisms [24]. Trans-UNet [25] was the first to incorporate a Vision Transformer (ViT) [26,27] into medical image segmentation by combining transformer and U-Net architectures. It integrates the self-attention mechanism of the transformer to capture global contextual information and enhance feature representation capabilities. Swin-UNet combines the advantages of the Swin transformer [28,29] and U-Net [17,30], introducing cross-layer communication mechanisms. This enables efficient feature information flow between different slices, thereby outperforming models based on the FCN method [16]. The Point-wise Multi-scale Fusion Network (PMF-Net) has been proposed to address these challenges by effectively integrating multi-scale features with a point-wise fusion mechanism. PMF-Net improves segmentation performance by capturing both fine-grained details and long-range dependencies, making it suitable for more complex medical image segmentation tasks, especially when contextual information is crucial [31,32]. Although deep learning technology has recently been applied to prostate image segmentation, its accuracy has not yet fully met clinical application requirements. Therefore, algorithms must be adjusted to address the issues of blurred boundaries and insufficient spatial information in the prostate MRI data. This study proposes a multi-scale context modeling-based U-Net (MCM-UNet) to effectively address the challenge of segmenting the prostate on T2W images. The main contributions of this study are as follows:

1. We introduce a novel multi-scale context modeling (MCM) module specifically designed for MRI prostate segmentation. This innovative module enhances pixel representation in boundary areas by minimizing the influence of irrelevant features, thereby improving segmentation results [33];
2. We employed a first-in-first-out (FIFO) strategy to dynamically adjust the dataset-level feature vectors and select the optimal ones. This strategy enhances the segmentation accuracy, especially in the challenging apex and base regions of the prostate;
3. We compiled data on 2175 prostate cases from 14 different local hospitals, constituting the largest private prostate dataset to date. The novelty, effectiveness, and robustness of the proposed model was validated using this dataset.

The remainder of this paper is organized as follows. Section 2 details the private prostate dataset and describes the prostate segmentation method. Section 3 describes the experimental setup and evaluates the performance and robustness of the proposed method on both private and public datasets using four assessment metrics. It also presents a visual analysis of the segmentation results and compares them with those of other mainstream segmentation methods. Section 4 discusses the innovations and limitations of the method, and Section 5 summarizes the contents of this article.

## 2. Dataset and Methods

### 2.1. Dataset

This retrospective study was reviewed and approved on 4 April 2023 by the Ethical Review Board of Huashan Hospital affiliated with Fudan University. Data were accessed for research purposes on 6 April 2023. These data were strictly anonymized during the collection process and personal information of participants was not available to the authors during the experiment, and the requirement of informed consent was therefore waived. All data for this retrospective study came from 2175 cases in 14 public hospitals in China from March 2012 to March 2022, including 984 scans of healthy prostates and 1191 scans of prostate cancer patients. This dataset is currently the largest known private T2W prostate dataset. All patients with prostate cancer underwent prostate biopsy and were diagnosed with prostate cancer. The pathological diagnoses were performed by hospital-certified pathologists using the Gleason grading system. The dataset includes patients aged 40–85 years, with an average age of 62 years, encompassing both healthy individuals and those at various stages of prostate cancer, from early to advanced stages. A detailed description of the data is presented in Table 1. Notably, Center-1, Huashan Hospital, which is affiliated with Fudan University and is one of China’s top public hospitals, served as our primary data source. All 984 healthy prostate scans originated from Center-1, and the 556 prostate cancer patient scans from this center constituted nearly half of all prostate cancer scans. The remaining 635 cases were sourced from 13 different hospitals that utilized various MRI machines and scanning parameters, thereby ensuring data diversity and verifying the robustness of the segmentation model. All the data were annotated by two experienced radiologists and reviewed by an authoritative imaging expert. The annotating physicians possessed 11 and 13 years of professional clinical diagnostic experience, respectively, while the reviewing expert possessed 31 years of professional experience. Prior to annotation, both experts underwent internships and task-specific training.

All data were independently annotated by two experts, and the correlation between their annotations was assessed using the correlation coefficient (CC) [31], which yielded a value of 0.97. For any annotations in which the correlation coefficient fell below 0.9, the reviewing expert conducted specialized quality control to ensure annotation accuracy and consistency. The correlation coefficient (CC) is defined as follows:

(1) $$CC_{i}=\frac{\sum_{i=1}^{n}\left(X_{i}-\bar{X}\right)\left(Y_{i}-\bar{Y}\right)}{\sqrt{\sum_{i=1}^{n}{\left(X_{i}-\bar{X}\right)}^{2}}\sqrt{\sum_{i=1}^{n}{\left(Y_{i}-\bar{Y}\right)}^{2}}}$$

where $X_{i}$ and $Y_{i}$ denote the annotations of the two experts in the prostate segmentation area in the *i*-th scan slice.

### 2.2. Methods

This section first introduces the overall structure of the proposed method, followed by descriptions of the context modeling module, dynamic feature storage module, and loss function. Finally, we describe the evaluation metrics.

#### 2.2.1. Architecture Overview

**Figure 2:**
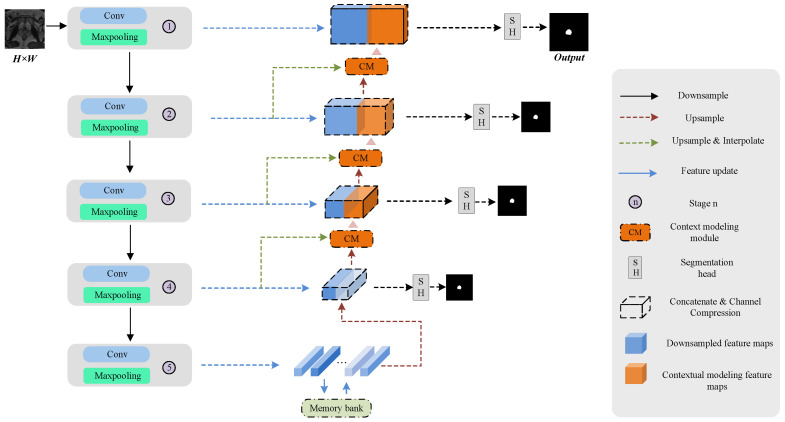
Overview algorithm framework of the proposed multi-scale context modeling-based U-Net (MCM-UNet) for prostate segmentation. The original T2W image dataset is fed into the encoder for high-level features. Then, the CM module captures the image-level features from multiple scales. Next, the encoder fuses the dataset-level features in the memory bank with the image-level features obtained after processing by the CM module to obtain global contextual features. Finally, the segmentation probability maps are obtained.

The proposed MCM-UNet, shown in Figure 2, consists of four main components: a feature encoding module, a context modeling (CM) module, a feature storage module, and a feature decoding module. Our focus is on accurately segmenting the prostate using MRI. The input to the network is a 3D T2W image of the prostate, set as $D=\left\{D_{1},\cdots ,D_{i},\cdots ,D_{n}\right\}$, where $D_{i}\in R^{H\times W}$ is the *i*-th slice, and *H* and *W* represent the size of the T2W image slices. The network outputs a probability map for prostate and background slices using nnU-Net as the backbone. As an enhanced version of U-Net, nnU-Net places more emphasis on image pre-processing. Through each encoding step, the network obtained feature maps with semantic information at various scales. To enhance the ability of the network to segment prostate boundaries, we integrated a CM module into every decoding step except for the final layer. Utilizing the deep supervision segmentation results and the upsampling outcomes from each layer of nnU-Net, we conducted attention computations to derive richer intra-image multi-scale contextual semantic features. In the final downsampling layer, we integrated a memory bank that dynamically retained intra-image semantic features from each batch by using a FIFO mechanism. We defined the collection of image-level semantic features within the memory bank as dataset-level semantic features. Combined with the CM module, the memory bank improves the model’s ability to extract intra-image contextual features and capture long-range semantic features across images, enhancing segmentation accuracy, particularly at the prostate apex and base. The CM module and the FIFO feature memory bank are detailed in Section 2.2.2 and Section 2.2.3, respectively.

#### 2.2.2. Context Modeling Module

**Figure 3:**
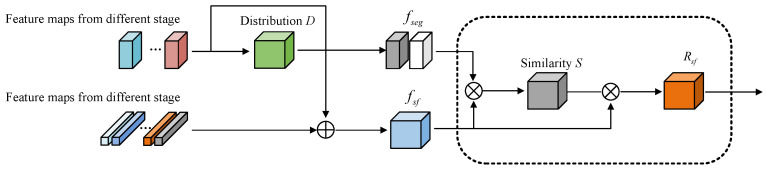
Image-level contextual feature acquisition process. The black dashed box part is the context modeling module.

The CM module refines pixel representation by extracting contextual semantic information in the image, minimizing irrelevant features, and improving boundary segmentation for a high-resolution output. As shown in Figure 3, the CM module has two inputs. We define the two inputs as $f_{seg}$ and $f_{sf}$, where $f_{seg}$ represents the deep supervision feature obtained from the category probability distribution *D*, which comprises two channels.

(2) $$f_{seg}=\sum_{n=1}^{N_{l}}\mathrm{Nor}(D_{l})\cdot R_{l},l\in \left\{0,1\right\}$$

where the size of $f_{seg}$ is H×W×C, H×W represents number of regional representations in the current stage, and *C* denotes the number of channels. *l* represents the category classification of the foreground and background images obtained by deep supervision. The size of $D_{l}$ is $N_{l}\times 1$, which represents the prediction probability of all pixels belonging to *l*, and Nor denotes the normalization function.

$f_{sf}$ refers to semantic features at the image level and represents the semantic characteristics of the current training image. We define $f_{sf_{j}}$ as the semantic features obtained at each upsampling stage, where $j\in \left[1,n\right]$ and *n* represents the number of the upsampling stage. When *j* = 1, $f_{sf_{1}}$ is the result of upsampling the features obtained from the last encoding layer and the result of feature merging and channel compression with the penultimate layer’s skip connection. When 1<j<n, $f_{sf_{j}}$ is the result of concatenate and channel compression of $f_{sf_{j-1}}$ with the skip connection features of the current stage.

Next, to reduce the impact of unrelated features, all pixels aggregate $f_{sf}$ and $f_{seg}$ together. The self-attention mechanism is then utilized to compute the similarity between $f_{sf}$ and $f_{seg}$:

(3) $$W_{sf}=\mathrm{Softmax}\left(\frac{f_{sf}^{HW\times C}⊗f_{seg}^{C\times HW}}{\sqrt{C}}\right)$$

where the size of $W_{sf}$ is HW×HW and ⊗ stands for matrix multiplication. Finally, the semantic features were aggregated based on similarity to obtain the image-level representation $R_{sf}$:

(4) $$R_{sf}=W_{sf}^{HW\times HW}⊗f_{sf}^{HW\times C}$$

In each skip connection, we perform identical steps to enhance the image-level features.

#### 2.2.3. First-in-First-Out Feature Update Strategy

**Figure 4:**
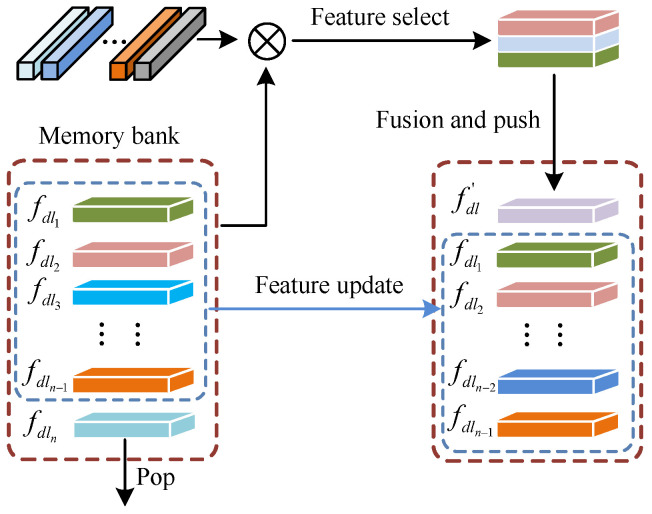
Schematic diagram of first-in-first-out-based memory bank operation mechanism.

Image-level semantic features alone lack robustness for current applications. Our network combines image-level and dataset-level features to improve the robustness and applicability of boundary region features. As shown in Figure 4, we defined the dataset-level semantic feature as $f_{dl}$, which represents the region derived from the training data across the entire dataset. $f_{dl}$ is derived from $f_{sf}$ and is more robust than $f_{sf}$ as it assimilates and continuously updates with more data to yield new dataset-level semantic features throughout the training process. Initially, we establish an N×H×W×C memory bank to store $f_{dl}$ dynamically, where *N* is the number of $f_{dl}$ and H×W×C is the size of the $f_{dl}$. Subsequently, we compute the similarity between the image-level semantic features $f_{sf}$ and $f_{dl}$, where $f_{sf}$ is obtained in the last stage of encoding and the $f_{dl}$ is obtained from the memory bank. We then select three $f_{dl}$ with the highest similarity with the input $f_{sf}$ in the memory bank and perform feature fusion on the three $f_{dl}$ and $f_{sf}$ to obtain a new $f_{dl}^{\prime }$:

(5) $$f_{dl}^{\prime }=\delta \left(f_{dl_{m}}⊕f_{dl_{n}}⊕f_{dl_{h}}⊕f_{sf}\right)$$

where ⊕ denotes the concatenation operation and δ is a transform function used to reduce the channels of the input matrix tensors. Simultaneously, the new $f_{dl}^{\prime }$ is pushed into the memory bank, and the $f_{dl_{n}}$ is popped out of the memory bank to complete the update of the feature container. This strategy enhances the extraction of semantic features at the dataset level, thereby improving segmentation of the apex and base of the prostate.

#### 2.2.4. Loss Function

During the training of our network, we utilized a combination of the Dice loss and cross-entropy loss as the loss function.

(6) $$L=L_{dice}+L_{CE}$$

We calculated the Dice loss for each sample in the batch and determined the average value for that batch, where the Dice loss is defined by the following formula:

(7) $$L_{dice}=-\frac{2}{\left|K\right|}\sum_{k\in K}\frac{\sum_{i\in I}u_{i}^{k}v_{i}^{k}}{\sum_{i\in I}u_{i}^{k}+\sum_{i\in I}v_{i}^{k}}$$

where *u* is the softmax output of the network and *v* is the one-hot encoding of the ground truth segmentation map. Both *u* and *v* have the shape I×K, with i∈I being the number of pixels in the training patch/batch and k∈K the class.

To evaluate the performance of our chosen loss function, we compared it to other commonly used loss functions, including focal loss and Tversky loss. Focal loss is particularly beneficial in cases of class imbalance, as it reduces the relative loss for well-classified examples and focuses more on hard-to-classify examples. Tversky loss, on the other hand, is specifically designed for handling imbalanced datasets and can be adjusted to emphasize either false positives or false negatives. However, despite their advantages, we found that the combination of Dice loss and cross-entropy loss yielded superior results in our specific task. The Dice loss emphasizes overlap, which is crucial for the accurate segmentation of medical images, particularly in small or irregularly shaped regions. The addition of cross-entropy loss further helps to fine-tune the boundary delineation by penalizing large discrepancies between predicted and true labels. This combination provides a balanced approach that not only improves segmentation accuracy but also ensures robustness in handling regions with unclear boundaries. Based on these findings, we believe that the combination of Dice loss and cross-entropy loss is well suited for our study’s objective of medical image segmentation.

#### 2.2.5. Evaluation Metrics

To comprehensively evaluate the performance of our proposed model, we utilized four performance metrics to assess the prostate segmentation results: the Average Symmetric Surface Distance (ASSD), 95% Hausdorff Distance (HD95), Jaccard index, and Dice similarity coefficient (DSC). Their definitions are as follows:

(8) $$\mathrm{ASSD}\left(A_{i},B_{i}\right)=\frac{1}{\left|S(A_{i})\right|+\left|S(B_{i})\right|}\times D$$

(9) $$D=\left(\sum_{a\in S(A_{i})}\min_{b\in S(B_{i})}\;\left|\left|a-b\right|\right|+\sum_{b\in S(B_{i})}\min_{a\in S(A_{i})}\;\left|\left|b-a\right|\right|\right)$$

where $A_{i}$ represents the ground truth of the prostate for the *i*-th sample, $B_{i}$ represents the corresponding output from the model, and ASSD is a measure of the average surface distance between the ground truth and segment outputs. The edge pixel set of $A_{i}$ is denoted by $S(A_{i})$, and the edge pixel set of $B_{i}$ is denoted by $S(B_{i})$.

(10) $$\mathrm{HD}95\left(X,Y\right)=\max\left\{95_{x\in X}^{th}d\left(x,Y\right),95_{y\in Y}^{th}d\left(X,y\right)\right\}$$

HD assesses the segmentation quality by calculating the maximum shortest distance between a point on the predicted contour and a point on the target contour. As HD is sensitive to outliers, we employed a more robust variant, HD95, which considers the 95th percentile instead of the absolute maximum. Thus, d(x,Y) is the minimum distance from the boundary pixel *x* to region *Y*.

(11) $$\mathrm{Jaccard}=\frac{\sum_{i=1}^{n}X_{i}⋂Y_{i}}{\sum_{i=1}^{n}X_{i}⋃Y_{i}}$$

The Jaccard index is commonly used to measure the accuracy of segmentation by quantifying the overlap between the predicted segmentation and the ground truth. Here, $X_{i}$ represents ground truth for the prostate of the *i*-th sample and $Y_{i}$ denotes the corresponding output from the model.

(12) $$\mathrm{DSC}=\frac{2TP}{FP+FN+2TP}$$

Among the overlap-based metrics, we utilize the well-known DSC, which ranges from 0% (no overlap) to 100% (complete overlap). Here, *TP* denotes true positives, *TN* denotes true negatives, *FP* denotes false positives, and FN denotes false negatives.

## 3. Experiments and Results

In this section, we describe the implementation of our experiment. We then present the results of our methods, offering both quantitative and qualitative analyses, and compare them with those of other methods. Finally, we conducted an ablation study to analyze the impact of different scenarios on network performance.

### 3.1. Implementation Details

To evaluate our methods, we used a substantial private dataset comprising 2175 MRI T2W scans and a public dataset known as PROMISE12 [34], which includes 50 MRI T2W scans. The PROMISE12 dataset, released for the MICCAI 2012 Prostate Segmentation challenge, serves as a well-established benchmark for evaluating prostate segmentation methods. The use of this publicly available dataset as an external validation set allows us to assess the generalization and transferability of our model to an independent dataset, thereby strengthening the credibility and robustness of our approach. Within the private dataset, 220 scans were used for external testing. The remaining 1955 scans were subjected to a 5-fold validation process, with the training validation set randomly and equally divided into five groups. In each fold, four groups were used for training, whereas the remaining group was used for validation.

For the implementation, we used a server equipped with a GeForce RTX 4090 GPU (NVIDIA, Santa Clara, CA, USA) with 24 GB of memory. All the experiments were conducted using the PyTorch 2.1.2 framework. MRI scans in the private dataset were interpolated to an isotropic voxel spacing of [0.66 × 0.66 × 5] mm^3^, followed by Z-score normalization. Subsequently, to train the 2D models, voxel patches were sliced along the axial direction to produce images of size 512 × 512, which served as input data. For the 3D models, each MRI scan was cropped to a voxel patch with dimensions of 320 × 320 × 16, centered around the prostate area.

All training sessions ran for a fixed duration of 1000 epochs, with each epoch comprising 250 training iterations, as recommended by the nnU-Net. The batch size was set to 12 for the 2D models and 2 for 3D models. The learning rate adhered to a ‘poly’ policy, decaying according to the specified formula ${\left(1-epoch/epoch_{\max}\right)}^{0.9}$ [35]. Stochastic gradient descent was employed as the optimizer, with the Nesterov momentum (μ) set to 0.99. Deep supervision was employed to enhance the training efficiency. The loss function combined binary cross-entropy loss and Dice loss in an equal weight (1:1 ratio).

### 3.2. Result Visualization

**Figure 5:**
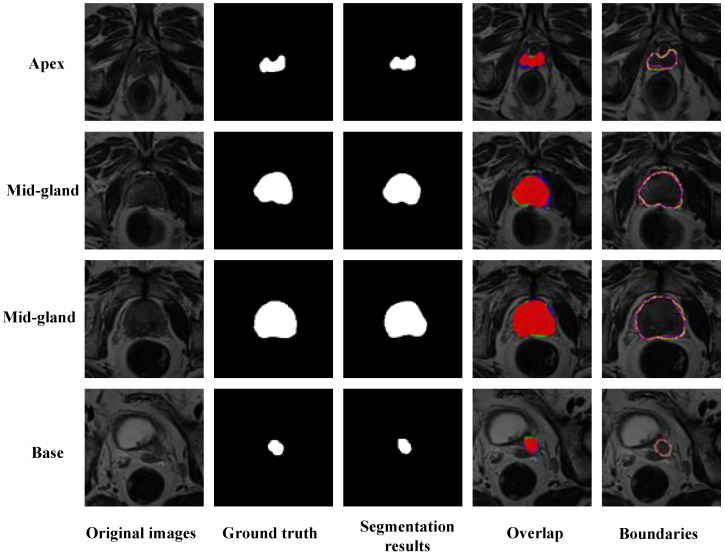
Visual presentation of the segmentation results of MCM-UNet. The first column shows the original images; the second column displays the ground truth annotations by radiologists. The third column presents the prostate segmentation results using our proposed MCM-UNet. The fourth column illustrates the overlap between the segmentation results and the ground truth, with red indicating areas of over-lap, green representing false positives, and blue indicating false negatives. The fifth column compares the boundaries of the segmentation results with the ground truth, where yellow lines represent the ground truth and purple lines indicate the segmentation outcomes.

Figure 5 shows the segmentation results of the proposed network for the test dataset. The first column of Figure 5 displays the original images, featuring four challenging cases that are difficult to discern, one apex area, two mid-gland areas, and one base area. The boundaries of the prostate were blurred, particularly in the apex and base areas, which were virtually indistinguishable to the naked eye. The second column presents the ground truth, which was manually annotated by the radiology experts. The third column displays the results segmented using MCM-UNet. The fourth column shows the overlap between the segmentation results and the ground truth, where red areas denote complete overlap, green represents false positives (indicative of under-segmentation), and blue signifies false negatives (indicative of over-segmentation). The fifth column compares the boundaries of the segmentation results with the ground truth; the yellow lines represent the ground truth, and the purple lines represent the segmentation outcomes.

**Figure 6:**
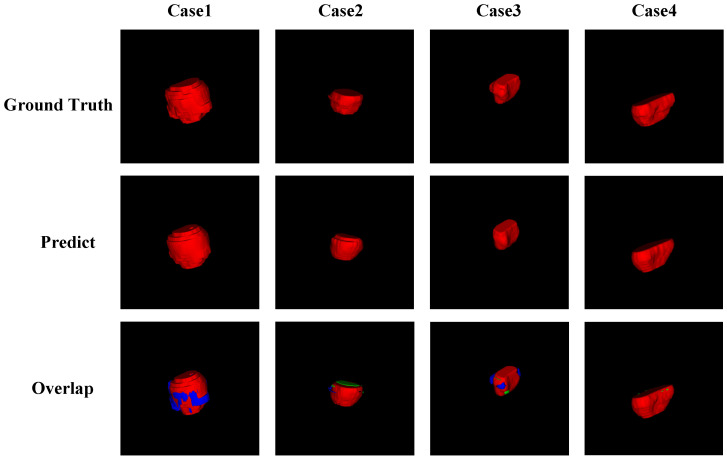
Visual presentation of the 3D segmentation results of MCM-UNet. The red region indicates perfect overlap, green represents under-segmentation, and blue signifies over-segmentation.

Figure 6 presents the 3D visualization results of the proposed method on the test dataset. The first row shows the ground truth, while the second row displays the 3D segmentation results of MCM-UNet. The third row illustrates the overlap between the segmentation results and the ground truth, where the red region indicates perfect overlap, green represents false positives (indicating under-segmentation), and blue signifies false negatives (indicating over-segmentation). From the segmentation results, it can be observed that MCM-UNet successfully segmented the entire prostate organ, with minimal occurrences of under-segmentation or over-segmentation, except in the peripheral areas.

### 3.3. Comparative Experiment

#### 3.3.1. Quantitative Analysis

To validate the effectiveness of our MCM-UNet, we conducted a comprehensive comparison with state-of-the-art 2D, 3D, and transformer-based medical image segmentation models. Specifically, our model was benchmarked against U-Net and U-Net++ for 2D segmentation, 3D-UNet [36] for 3D segmentation, and Swin-UNet and Trans-UNet for transformer-based segmentation. In additional, nnU-Net served as the baseline for comparison. All models, except nnU-Net, were trained from scratch under conditions identical to those used for MCM-UNet.

The quantitative results presented in Table 2 highlight the superior performance of MCM-UNet over other models. On private datasets, ASSD, HD95, Jaccard index, and DSC for our model were 0.58 voxels, 1.80 voxels, 83.17%, and 91.71%, respectively. Compared to the baseline nnU-Net, our model shows improvements of 0.43 voxels in ASSD, 4.38% in Jaccard index, and 3.58% in mean DSC. On the PROMISE12 prostate segmentation task, our method also demonstrated improvements across all metrics of 0.98 voxels, 2.72 voxels, 3.38%, and 1.15%, respectively.

The CM module and memory bank are crucial in extracting and leveraging both image-level and dataset-level contextual features, enhancing the model’s effectiveness, particularly at the prostate boundary and challenging apex and base areas. This strategic integration significantly boosts the performance across key metrics, underscoring the advanced capabilities of our MCM-UNet in handling complex segmentation tasks.

#### 3.3.2. Qualitative Evaluation

**Figure 7:**
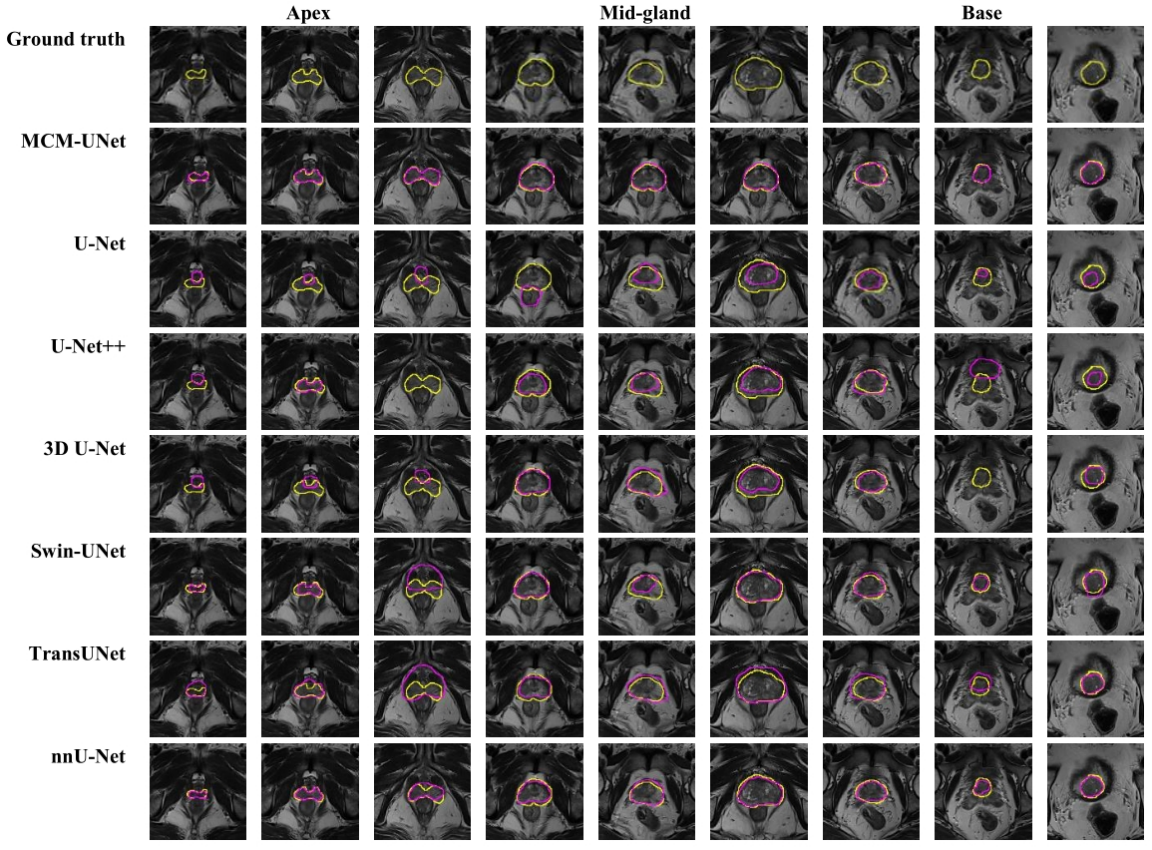
Visualization comparison of segmentation results of different methods in different parts of the prostate. The yellow lines and purple lines are the ground truth and segmentation results, respectively. Each column is a 2D slice image of different samples, where the first three columns represent the apex parts of the prostate, the middle three columns are the mid-gland, and the last three columns represent the base parts of the prostate. Each row is the segmentation results of different methods. From top to bottom: ground truth, our method, U-Net, U-Net++, 3D U-Net, Swin-UNet, Trans-UNet, nnU-Net.

Figure 7 shows the representative results of the proposed MCM-UNet and comparison methods, highlighting its superior accuracy and consistency. Basic models like U-Net, 3D U-Net [36], and U-Net++ faced significant challenges with under-segmentation, particularly in the apex and base regions, where anatomical structures such as the vas deferens were frequently misidentified as the prostate. These models also struggled with class imbalance, as they were not robust enough to handle the varied intensity distribution and small size of prostate structures in certain regions. Furthermore, U-Net and its variants suffered from boundary ambiguity, especially in regions with blurred or poorly defined margins. Swin-UNet and Trans-UNet, while incorporating transformer architecture for better context learning, still showed performance gaps in the apex and base regions. These models also displayed a sensitivity to computational efficiency, as the added transformer layers significantly increased training time and memory consumption without a proportional improvement in segmentation accuracy. nnU-Net, as our baseline, performed relatively well, but still struggled to accurately delineate detailed prostate boundaries, especially in regions with low contrast. In contrast, the proposed MCM-UNet addresses these limitations by effectively utilizing both intra-image and inter-image contextual information, which enables precise segmentation even in regions with unclear boundaries. Furthermore, our model is more robust to class imbalance and computationally efficient, providing superior segmentation across all regions, regardless of prostate shape variations.

### 3.4. Ablation Study

#### 3.4.1. Hyper-Parameter Ablation Study

In our designed network structure, there are two important hyper-parameters: the number of CM modules *n*, and the number of features stored in the memory bank *m*. To study the effects of different parameter settings on the segmentation performance of the network, we set different parameter values to train the network. As shown in Table 3, we set n to one and six. The value of m is 32, 64, 128. We used HD95 and DSC as evaluation metrics to discuss the effects of the parameters on the prostate model. Table 3 shows that when *n* is 6 and *m* is 64, our proposed model obtains the lowest HD95 value and the highest DSC value for prostate segmentation.

#### 3.4.2. Network Structure Ablation Study

We conducted an ablation study, as detailed in Table 4, utilizing our private dataset. To ascertain the contributions of the proposed CM module and FIFO feature update strategy with the memory bank (MB), we disabled various components within the entire network and trained the models accordingly. According to Table 4, integrating the CM module into nnU-Net improved the DSC from 88.13% to 88.62%, an increase of 0.49%. Similarly, the FIFO update strategy with the MB enhanced the DSC to 89.21%, indicating an increase of 1.08%. Furthermore, when both the CM module and MB were added to U-Net, the DSC increased to 91.71%, which is an increase of 3.58%. This highlights the significant positive impact of both the CM module and the MB on segmentation performance, particularly noting the enhanced improvements through their synergistic utilization. While the CM module enhances the segmentation of the boundary regions, the MB’s capability to extract dataset-level semantic features substantially benefits the network, especially in improving segmentation at the apex and base of the prostate.

## 4. Discussion

In this study, we enhanced the network architecture based on classical U-Net and assessed its performance in segmenting prostate MRI T2W images. Accurate segmentation of the prostate is crucial for effective prostate cancer treatment, and provides radiologists with essential indicators for diagnosis and prognosis. We propose a network that utilizes multi-scale context modeling to optimize the extraction of contextual features within each image layer. By integrating a CM module into each layer of skip connections, we reduced the influence of irrelevant features on the segmentation outcomes and enhanced the boundary pixel representation. Additionally, a storage module was incorporated to dynamically store feature vectors through a FIFO mechanism, facilitating the capture of inter-image features and enhancing the segmentation of critical prostate regions, particularly the apex and base. As shown in Table 2 and Figure 7, our method achieves a more accurate segmentation than other common methods and excels in all evaluation metrics.

Currently, most prostate segmentation methods are trained and validated on public datasets or small-scale private datasets, typically containing fewer than 100 samples, such as PROMISE12 and MSD prostate. These datasets are generally limited in size and lack representation for patients with prostate cancer. We collected 2175 T2W MRI scans of the prostate from 14 hospitals, encompassing both healthy individuals and patients with prostate cancer, which were accurately annotated by experienced radiologists. This marks the first instance in which prostate segmentation has been performed and validated on such a large dataset, enhancing the generalization and robustness of our method.

In this study, we utilized the CM module, which enhances the pixel representation of boundaries and reduces the influence of irrelevant features on segmentation outcomes. We investigated the impact of the number of CM modules on performance by comparing the addition of a single CM module to integrating CM modules in every layer through hyper-parameter experimentation. We discovered that layer-by-layer addition not only yields the highest segmentation accuracy but also increases the computational time by only 10% compared to a single-layer addition, a marginal increase. Consequently, we adopted a layer-by-layer addition of CM modules to enhance the segmentation accuracy of the model for the prostate region. Another hyper-parameter in our method is the size of the memory bank, which determines the number of stored image features. According to Table 3, a memory bank size of 64 offers optimal performance without excessive computational demand. Increasing the size to 128 or 256 does not significantly enhance performance but does lead to higher computational loads and reduced training efficiency. This effect occurs because the median number of prostate image layers is 18, allowing the size 64 memory bank to accommodate nearly four groups of distinct data. Larger sizes such as 128 and 256 can store 7 and 14 groups, respectively, but increase computation time during feature similarity calculations and are inefficient at managing long-distance feature relations. Therefore, a memory bank size of 64 is deemed most appropriate.

We trained the model on a private dataset and conducted external validation on both this dataset and the open-access PROMISE12 dataset, achieving favorable results in both cases. When examining Table 2, it is evident that the same method yields different performances on various datasets for HD95 and ASSD. The metrics are higher on PROMISE12 not due to poor model generalization, but because PROMISE12 comprises thin-sliced data with a larger slice count. This affects the HD95 and ASSD performance, though the DSC values remain consistent. In subsequent work, we will incorporate a greater volume of thin-slice data to enhance the diversity of the experimental datasets.

In this study, we focused solely on the T2W modality. However, employing multiparametric MRI, which includes T1-weighted (T1W) images, may enhance prostate segmentation accuracy by providing details that T2W images do not capture. In future studies, we plan to introduce multiparametric MRI images as separate input channels for the network, with each channel representing a different MRI modality. It is important to note that T1W typically offers poor contrast to the prostate tissue. Consequently, channel weighting is crucial during model training to optimize performance.

Regarding the clinical applicability of our method, we foresee several steps necessary to integrate it into clinical environments. First, the model would need to be validated on a diverse set of clinical datasets to ensure its robustness across different populations and scanner configurations. Additionally, integration with existing clinical systems, such as Picture Archiving and Communication Systems (PACS), is essential for seamless usage by radiologists. Real-time processing is another crucial consideration, as clinical workflows demand fast and accurate results. Thus, optimizing the model for inference speed without sacrificing accuracy is a key area for future work. Finally, user interface (UI) considerations are vital for clinical adoption. The model should be incorporated into a user-friendly platform that allows radiologists to easily visualize the segmentation results, make adjustments if necessary, and incorporate the model’s output into their diagnostic process. Designing an intuitive and efficient UI will be essential for ensuring that the tool enhances, rather than disrupts, the workflow in clinical settings.

## 5. Conclusions

In this study, we introduce a novel network: the multi-scale context modeling module-based UNet (MCM-UNet). This network adopts a multi-scale optimization strategy by integrating a CM module into each skip connection. The CM module enhanced pixel representation in boundary regions by selectively reducing irrelevant features. Furthermore, by employing a FIFO update strategy, feature vectors are dynamically adjusted to capture dataset-level semantic features. Our MCM-UNet demonstrated significant improvements in segmentation performance, notably in boundary regions and in the apex and base regions of the prostate. We trained and evaluated the model using 2175 high-quality clinical prostate images, yielding precise segmentation results. This model provides a reliable tool for enhancing the accuracy of radiation therapy in prostate cancer treatment.

## Figures and Tables

**Table 1:** The hospitals, MR scanners, and acquisition parameters of the data. * denotes that the case from the center consisted of a healthy prostate.

Muticentre	MR Scanner	Shape	Spacing (mm^3^)	FOV (mm^3^)	Training Set	External Test Set	Total Cases
Center-1 *	GE SIGNA EXCITE	512 × 512 × 16	0.625 × 0.625 × 6	320 × 320 × 96	1386	154	1540
	GE Signa HDxt	512 × 512 × 16	0.586 × 0.586 × 7	300 × 300 × 112			
	GE Discovery MR750	512 × 512 × 16	0.547 × 0.547 × 4	280 × 280 × 64			
	SIEMENS Verio	256 × 256 × 20	0.781 × 0.781 × 3.6	200 × 200 × 72			
Center-2	SIEMENS Skyra	640 × 640 × 20	0.312 × 0.312 × 3.6	200 × 200 × 72	48	6	54
Center-3	Philips Ingenia	480 × 480 × 25	0.375 × 0.375 × 3.85	180 × 180 × 96	47	5	52
	SIEMENS Avanto	512 × 488 × 25	0.429 × 0.429 × 3.6	220 × 210 × 90			
Center-4	SIEMENS Skyra	640 × 640 × 24	0.359 × 0.359 × 5.5	230 × 230 × 132	60	7	67
Center-5	SIEMENS Skyra	640 × 640 × 20	0.375 × 0.375 × 4.2	240 × 240 × 84	84	9	93
Center-6	GE Signa HDxt	512 × 512 × 17	0.586 × 0.586 × 4.3	300 × 300 × 73	43	5	48
	Philips Ingenia	480 × 480 × 20	0.437 × 0.437 × 3.3	210 × 210 × 66			
Center-7	UIH uMR uMR560	384 × 384 × 21	0.52 × 0.52 × 3.6	200 × 200 × 76	16	2	18
Center-8	GE Signa	512 × 512 × 22	0.391 × 0.391 × 3.5	200 × 200 × 77	48	6	54
	GE Discovery MR750w	512 × 512 × 24	0.469 × 0.469 × 3.5	240 × 240 × 84			
Center-9	GE Discovery MR750w	512 × 512 × 12	0.391 × 0.391 × 4.8	200 × 200 × 58	46	5	51
	SIEMENS TrioTim	320 × 320 × 16	0.719 × 0.719 × 4.4	230 × 230 × 70			
Center-10	GE Signa HDxt	512 × 512 × 20	0.566 × 0.566 × 4.4	290 × 290 × 88	12	2	14
Center-11	GE Signa HDxt	512 × 512 × 24	0.508 × 0.508 × 6.0	260 × 260 × 144	47	6	53
Center-12	GE Signa HDxt	512 × 512 × 20	0.469 × 0.469 × 4.0	240 × 240 × 80	44	5	49
Center-13	SIEMENS Skyra	320 × 320 × 20	0.75 × 0.75 × 3.85	240 × 240 × 77	57	6	63
Center-14	SIEMENS Prisma	320 × 320 × 30	0.812 × 0.812 × 5.2	260 × 260 × 156	17	2	19
	UIH uMR 770	576 × 576 × 24	0.417 × 0.417 × 6	240 × 240 × 144			

**Table 2:** Quantitative performance comparison of our method with classic medical image segmentation network on private and PROMISE12 datasets.

Method	Private				PROMISE12			
	ASSD (voxel)	HD95 (voxel)	Jaccard (%)	DSC (%)	ASSD (voxel)	HD95 (voxel)	Jaccard (%)	DSC (%)
U-Net [17]	5.07	18.86	61.85	76.43	2.61	7.89	70.45	81.34
U-Net++ [22]	0.81	2.82	64.79	78.63	1.71	6.76	69.62	80.20
3D U-Net [36]	0.79	1.41	68.37	81.21	1.95	6.68	71.51	82.91
Swin-UNet [28]	0.85	2.23	75.24	85.87	1.32	4.73	70.89	82.43
Trans-UNet [25]	0.83	3.31	71.01	83.05	1.51	6.86	72.74	83.72
nnU-Net [20,21]	1.01	1.73	78.79	88.13	2.05	5.74	78.20	89.32
MCM-UNet	0.58	1.80	83.17	91.71	1.07	3.02	81.58	90.47

**Table 3:** Impact of hyper-parameter settings on network performance (HD95 and DSC as evaluation metrics).

Parameters		Prostate Segmentation		
***m***	***n***	**HD95 (voxel)**	**DSC (%)**	**Times (epoch/s)**
1	32	3.86	89.27	33.1
6	32	1.98	91.24	35.3
1	64	3.52	90.32	34.2
6	64	1.80	91.71	37.6
1	128	3.14	90.25	36.4
6	128	2.82	91.42	40.5

**Table 4:** Ablation study of comparison with baseline.

Backbone	CM	MB	DSC (%)
nnU-Net			88.13
nnU-Net	✔		88.62
nnU-Net		✔	89.21
nnU-Net	✔	✔	91.71

## Data Availability

Data cannot be shared publicly due to patient privacy reasons. The data are available from the Ethics Committee of Huashan Hospital affiliated to Fudan University (contact via Prof. Daoying Geng) and are suitable for researchers who meet the criteria for accessing confidential data.

## Abbreviations

The following abbreviations are used in this manuscript:

DRE	Digital Rectal Examination
PSA	Prostate-Specific Antigen
T2W	T2-Weighted
MRI	Magnetic Resonance Imaging
DWI	Diffusion-Weighted Imaging
DCE	Dynamic Contrast-Enhanced
EBRT	External Beam Radiation Therapy
CNN	Convolutional Neural Networks
FCN	Fully Convolutional Network
ResNet	Residual Network
ViT	Vision Transformer
PMF-Net	Multi-Scale Fusion Network
CC	Correlation Coefficient
T1W	T1-Weighted
UI	User Interface
PACS	Picture Archiving and Communication Systems
